# Hindsight: Review of Preclinical Disease Models for the Development of New Treatments for Uveal Melanoma

**DOI:** 10.7150/jca.53954

**Published:** 2021-06-04

**Authors:** Caoimhe Goldrick, Letizia Palanga, Bobby Tang, Grace Mealy, John Crown, Noel Horgan, Susan Kennedy, Naomi Walsh

**Affiliations:** 1 National Institute for Cellular Biotechnology, School of Biotechnology, Dublin City University, Dublin, Ireland.; 2 Royal Victoria Eye and Ear Research Foundation, Royal Victoria Eye and Ear Hospital, Adelaide Road, Dublin, Ireland.; 3 Department of Medical Oncology, St. Vincent's University Hospital, Dublin, Ireland.

**Keywords:** uveal melanoma, preclinical disease models, personalised medicine, cell lines, PDX, GEMM, zebrafish models

## Abstract

The molecular, histopathological, genomic and transcriptomic characteristics of uveal melanoma (UM) have identified four molecular subgroups with different clinical outcomes. Despite the improvements in UM classification and biological pathology, current treatments do not reduce the occurrence of metastasis. The development of effective adjuvant and metastatic therapies for UM has been slow and extremely limited. Preclinical models that closely resemble the molecular and genetic UM subgroups are essential for translating molecular findings into improved clinical treatment. In this review, we provide a retrospective view of the existing preclinical models used to study UM, and give an overview of their strengths and limitations. We review targeted therapy clinical trial data to evaluate the gap in the translation of preclinical findings to human studies. Reflecting on the current high attrition rates of clinical trials for UM, preclinical models that effectively recapitulate the human *in vivo* situation and/or accurately reflect the subtype classifications would enhance the translational impact of experimental data and have crucial implications for the advancement of personalised medicine.

## Introduction

Despite being a rare subtype of melanoma (approximately 3% of melanoma cases), uveal melanoma (UM) is the most common intraocular cancer in adults 1. Approximately 50% of UM patients develop metastases within 10 years of diagnosis, with the liver being the most common site of metastasis, followed by lungs and bone 1. Metastatic UM (mUM) patients have a median overall survival of 6 to 12 months 2,3, and while effective localised treatments exist to target the primary tumour, there are no current therapies available to cure patients at metastatic stages 4. Therefore, generating preclinical models to better characterise the genetic landscape and evolution of UM is critical in order to identify new cancer biomarkers and therapies. This review will assess current preclinical models of UM and their merits in furthering the development of effective treatment strategies. In doing so, we provide a comprehensive overview of targeted therapies in clinical trials for UM and link preclinical research outcomes with clinical results.

### Clinical epidemiology of uveal melanoma

Several variables may alter the incidence of UM, such as race, sex, and age. UM occurs most frequently in the Caucasian population, followed by Asian, Hispanic, and Black populations. This is likely to relate to physical features, including pale skin and light eye colour, which are known risk factors in the development of UM 5. In Ireland, the age-adjusted incidence of uveal melanoma at 9.5 cases per million is one of the highest in the world 6. UM most commonly arises in the choroid (90%), followed by the ciliary body (6%) and iris (4%) 7. Tumours are histopathologically characterised by the morphology of the dominant cell type, including spindle, epithelioid, mixed, and necrotic cell types. Mixed cell type consists of varying amounts of spindle and epithelioid cells, while necrotic cell type, as the name suggests, is predominantly made up of dead cells of unidentifiable morphology 8. Prognosis is determined by the analysis of tumour type, cellular morphology, mitotic figures, cytogenetic aberrations, tumour diameter, and the presence of metastases. Survival rates are highest for spindle cell-type tumours, followed by mixed, and necrotic, with tumours of epithelioid morphology having the poorest outcomes 2,9,10.

### Genetic landscape of uveal melanoma

Understanding the molecular and genetic landscape of UM is vital in deciphering its pathogenesis and determining patient prognosis. While clinical features, such as tumour size, histopathological state, and location in the uvea can indicate the presence of metastatic risk, cytogenetic and molecular profiling, such as fluorescence *in situ* hybridisation (FISH) studies, can depict disease state accurately 11. A patient's disease status can be classified as either low or high-risk. The former is a less aggressive form of UM, rarely leading to metastasis, while the latter features cancer stem-cell like cells and an aggressive, commonly metastatic disease 12.

Cytogenetic alterations involve losses relating to chromosomes 1p, 3, 6q and 8p, and amplifications relating to 6p and 8q are considered as UM biomarkers 13,14. TCGA global and integrated molecular and genomic analyses classifies UM into four molecularly distinct, clinically relevant subtypes 15. Monosomy 3 and 8q gain are particularly associated with a poor disease prognosis and metastatic state 13. Conversely, disomy 3 is associated with low risk UM, and thus, low rates of metastasis.

An early event in primary UM resulting from genetic mutations is activation of G protein-coupled receptor signalling. This occurs through mutually exclusive mutations in *GNAQ* or* GNA11*, or via rare mutations in *CYSLTR2*, and/or *PLCB4* 16,17. *GNAQ* and *GNA11* encode α subunits of Gα_q/11_ heterotrimeric G proteins, respectively. Abnormalities here are the most prominent initiating mutations in UM, arising in 90% of cases 18,19. Such mutations alter Gln209 and Arg183, inhibiting GTPase activity, causing constitutive activation of G proteins 15. Related downstream signalling pathways include MAPK, PI3K/Akt and Rho GTPase signalling, all of which are a large research focus in the field.

Although less prominent than *GNAQ* and *GNA11* mutations, mutations in the cysteinyl-leukotriene receptor 2 (*CYSLTR2*) gene occur in 4% of primary UM, and also lead to the constitutive activation of G_αq_ proteins relating to *CYSLTR2*. The downstream effects of this abnormality were found to promote tumorigenesis *in vivo* 19. Gain of function mutations in phospholipase β4 (*PLCβ4*) can also occur in UM, at a rate of 2.5-4% and similarly, enhances GPCR signalling 18. *PLCβ4* can act as a downstream target of Gα signalling, and namely the canonical target of *GNAQ* and *GNA11* gene products. *PLCβ4* hotspot mutation at amino acid residue, D630, specifically activates the PLC-PKC-MAPK signalling pathway downstream of *GNAQ* signalling, implying that this may be a pathway of particular importance in UM 18. The MAPK signalling pathway is integral in growth regulation, stimulation of pro-proliferative and anti-apoptotic genes, indicating these mutations may confer a key role of Gαq signalling in the pathogenesis of UM 20.

The Breast Cancer 1 (BRCA1)-associated protein 1 (*BAP1*) is a tumour suppressor gene which is inactivated in 70% of metastatic UM. Consequently, *BAP1* inactivation is associated with high risk UM 21. *BAP1* is found on chr 3p21.2, anomalies of which strongly correlate with monosomy 3 22. *BAP1* promotes DNA repair, regulates transcription, cell differentiation, and cell death 23. Owing to its role in DNA repair, loss of *BAP1* function may contribute to the emergence of cytogenetic alterations later in UM progression.

Mutations in eukaryotic initiation factor 1A (*EIF1AX*) are thought to occur in cases of disomy 3, rarely presenting in monosomy 3 tumours, consolidating its relatively low metastatic risk 24-26. This gene plays a role in initiation of translation, which is altered by arising mutations.

Mutations in splicing factor 3B subunit 1 (*SF3B1*) and serine and arginine rich splicing factor 2 (*SRSF2*) occur in intermediate risk tumours 22,27.* SF3B1* and *SRSF2* encode spliceosomal proteins, mutations of which culminate in a large number of splice variants in UM. The precise effects of these abnormalities in UM remain elusive, however, in other cancers, these mutations can reduce DNA damage repair, and are therefore potential contributors to emerging cytogenetic mutations 28,29. These aforementioned mutations of *BAP1*, *EIF1AX*, *SF3B1* and *SRSF2*, tend to be mutually exclusive, providing effective molecular biomarkers of metastatic outlook using techniques such as gene expression profiling (GEP).

## *In vitro* uveal melanoma cell line models

### Established UM Cell Lines: 2D and 3D models

Experimental use of cell lines is a convenient and cost-effective method of carrying out preliminary research. Cell lines have been shown to accurately recapitulate the key genomic events of many types of cancer, including breast cancer and ovarian cancer 30,31. Prior to the establishment of UM cell lines, cutaneous melanoma cell lines presented an attractive alternative. However, genetic discrepancies may have added to the lack of progression in positive research outcomes during this time, as numerous cell lines, such as OCM-1, OCM-3, and OCM-8, which were initially thought to be of UM origin have been found to be misidentified derivatives of melanoma cell lines 32,33. Although a subtype of melanoma, UM presents a distinct genetic landscape to cutaneous melanoma. For example, while many cutaneous melanoma cell lines characteristically harbour a mutation in the *BRAF* gene, this aberration does not commonly arise in UM 34. OCM-1, OCM-3 and OCM-8 each present with V600E mutation, with the addition of V599E in the case of OCM-1. Perhaps this discrepancy was, in part, responsible for lack of substantial progression in understanding UM pathogenesis and development of novel therapeutics prior to UM cell lines being readily available.

Therefore, accurately modelling the genetic landscape of UM is fundamental to advancements in preclinical research. In recent years, there has been a growing repertoire of established UM cell lines derived from primary and secondary patient tumours, or patient-derived xenografts (Table 1). Most of the established UM cell lines harbour at least one known UM driver mutation, and may mirror a proportion of the genetic landscape UM. Consideration of patient clinical and histopathological characteristics is also of importance in drawing connections with underlying genetic features and formulating a complete cell line profile 35. Cell lines remain an established first line model in carrying out preliminary preclinical experimentation. In recent years, there have been tremendous initiatives to improve the library of UM cell lines in terms of capturing the mutational signature of UM. In general, most *in vitro* studies assessing the progression, development and treatment response of UM are performed using 2D UM cell lines. However, consideration needs to be given in the experimental design when using such models. *In vitro*, 2D culture of cells is restricted in its ability to mirror cellular interactions with the extracellular matrix, which is an important aspect in understanding tumorigenesis and cancer progression. The need for more complex cellular systems is required to further understand the development of this disease, and to accurately represent drug efficacy in UM patients.

Cell lines can also be used to generate 3D *in vitro* models, which offer a number of advantages over 2D cell culture, such as the establishment of living biobanks and genomic analysis. In comparison to 2D models, 3D cell culture models can provide a more realistic model of how therapeutic treatments take effect in cancer cells and allow for exploration of alternative treatments. Several studies have examined the use of 3D spheroids as models of UM, using established and primary UM cell lines. Recently, Aughton et al. assessed the ability of established UM cell lines (92.1, OMM-2.5, MM66, MP41, OMM-1, and MP46) and primary UM tissue to form spheroids. This study highlighted the utility of UM cell line spheroids in *in vitro* drug screening assays. Compared to 2D cell lines, the 3D cell line spheroids exhibited altered drug responses, with increased resistance to doxorubicin and higher sensitivity to treatment with selumetinib 48. This study suggests the importance of 2D vs 3D culture drug efficacy studies, as drug penetration, different metabolic states, and cell cycle growth rate, are more likely to affect the predicted preclinical efficacy. Other studies have also shown the ability of UM cell lines to be cultured as 3D spheroids. Goncalves et al. embedded cell lines 92.1, MEL270 and MEL290 in collagen and demonstrated MEKi cytotoxicity 49. The morphological nature of 3D cell culture makes it an excellent model for studying metastasis of UM also. Migration of UM cancer cells in 3D culture shows a more natural and realistic path than would be observed in 2D culture. Fornabaio et al. studied extravascular migratory metastasis and angiotropism in both cutaneous and uveal melanoma using 3D cell culture. While the most common metastatic path for tumour cells to spread occurs through intravascular or intralymphatic routes, metastasis may also occur through extravascular migratory metastasis, as with angiotropism. The UM cell lines OMM 2.3-GFP tagged and OMM 2.5-GFP tagged were added to a plate coated with basement membrane extract containing human endothelial HUVEC cells, which developed tubular structures. Real-time imaging of the 3D Matrigel-embedded UM cell line co-cultures found that both OMM-2.3-GFP and OMM-2.5-GFP showed migration of uveal melanoma cells along, and towards, endothelial cells, and subsequent attachment to the endothelial tubules. This provides evidence of angiotropism of uveal melanoma cells in endothelial tubules in 3D cultures 50. Such studies suggest that 3D cell culture may be advantageous over 2D studies in some settings, and may provide a more accurate means to predicting *in vivo* tumour responses.

## Mouse models of UM

### Xenograft mouse models

Xenografts are a valued tool in cancer biology, providing an efficient model for exploring the *in vivo* tumour microenvironment, and drug screening. Such models can be generated using established cell lines, primary tumours, or secondary tumour metastases, such as from the liver, largely maintaining the genetic characteristics of the original patient tumour. Cells can be injected into selected compartments of the eye to mimic specific primary tumours, intravenously, or at secondary tumour sites to study metastases. This has been demonstrated in a growing number of studies, using a variety of established UM cell lines. For example, in assessing novel UM biomarkers, Barisione et al. carried out intrasplenic injection of either 92.1 or MEL270 cells into 6NU/NU and 9NOD/SCIDIL2Rγnull (NOG) mice 51. Similarly, 92.1 or MEL202 have been inoculated subcutaneously into hIL2-NOG mice for use in exploring the *in vivo* efficacy of HER2 CAR-T cells in eradicating UM 52. Metastatic cell line OMM-1 has been injected subcutaneously into NOD/SCID mice, and the resulting models used to evaluate the anti-tumour activity of niclosamide, preclinically 53.

However, the ability of cell line xenografts to recapitulate the genetic and molecular landscape of the patient tumour from which they were derived may be unsatisfactory, due to changes that occur through *in vitro* culturing. Established cell lines often lack some of the mutational burden present in the tumour of origin, such as cell lines MEL285 and MEL290 (Table 1). This discrepancy in recapturing the original genomic signature using cell line implants, gives way to an attractive alternative; patient-derived xenograft (PDX) models. These models have repeatedly been observed to retain the mutational burden, chromosomal abnormalities, and histopathological features of the original tumour. Successful generation of PDX models can also provide a means for establishing new cell lines from the animal (Table 2).

Némati et al. established 16 PDX mouse models, using severe-combined immunodeficient (SCID) mice. Chromosome 3 status was examined as a method of assessing genomic correlation between xenograft tumours and patient tumours via single nucleotide polymorphism (SNP) array analyses. Results showed that in the case of disomic, heterozygous, and monosomic chromosome 3, status of xenografts corresponded to that of the tumour of origin. However, chromosome 3 isodisomic patient tumours gave rise to monosomic xenograft tumours 54.

Similarly, PDX models can be of use in studying metastasis in UM. Kageyama et al. generated an orthotopic PDX mouse model from UM liver metastases. In examining five key mutations in UM (*GNAQ, GNA11, BAP1, SF3B1* and *EIF1AX*), the xenograft tumours matched the genomic signature of the original patient tumours in 83% of cases. Furthermore, features such as histological characteristics and DNA copy number variations of the tumour of origin were retained in the xenograft tumours 55.

UM PDX can be used as preclinical models in identifying novel therapeutic targets and advancing treatment strategies. Carita et al. demonstrated therapeutic efficacy for combination of PKC/mTOR inhibitors with PKC/p53-MDM2 inhibitors or mTORC1 inhibitors, both of which resulted in tumour regression in PDX mouse models by targeting the PKC pathway downstream of *GNAQ* and *GNA11* driver mutations 56. However, although PDX mouse models of UM are effective in modelling UM *in vivo* while maintaining many fundamental facets of the disease as it appears in humans, a consideration to this method is the need to suppress the mouse immune system to ensure the graft is accepted by the host. This limits the accuracy in studying the relationship between the tumour and its microenvironment in such models, as the involvement of the host immune response is diminished. This, consequently, hampers their use in studying immunotherapies. The cost and the time to initiate such models add additional limitations. Despite this, xenograft models present a useful tool in the preclinical setting for non-immunologic agents.

### Genetically Engineered Mouse Models (GEMMs)

A major attraction to the use of genetically engineered mouse models (GEMMs) is that it encompasses native tumorigenesis in an immunocompetent host, mimicking the *in vivo* tumour microenvironment and stages of tumorigenesis. Fundamentally, this approach allows observation of specific effects of mutations on oncogenic signalling.

Prior to extensive study of the UM genome, some earlier GEMMs included tumours that originated in the retinal pigment epithelium, as opposed to the uveal tract 57. Many of these earlier models are driven by molecular changes which do not occur in UM, therefore, limiting their clinical relevance (Table 2). Since the establishment of *GNAQ/GNA11* as key oncogenic drivers in a large cohort of UM, genetically engineered overexpression of *GNAQ*^Q209L^ in mice deficient for p16^Ink4a^ and p19^Ink4b^ have been developed which demonstrate elevated *YAP* signalling downstream of oncogenic *GNAQ*. However, there was a high incidence of cutaneous melanoma, as opposed to UM in these models. While thought to underlie early events in UM tumorigenesis, *GNAQ/GNA11* mutations alone are insufficient to recreate the UM phenotype in this model. In fact, uvea nevi which are not always precancerous are commonly present with GNAQ/GNA11 mutations 58,59. In the case of *GNA11* mutations, overexpression of *GNA11*^Q209L^ at a conditional knock-in allele caused sporadic UM development in GEMMs. While the combination of *BAP1* deletion with *GNAQ11*^Q209L^ yielded larger cutaneous melanocytic lesions, there was no such observed effect in UM tumours 60. This may indicate that other genetic cues may be required to replicate the aggressive phenotype associated with *BAP1* anomalies in humans.

Tolleson et al. generated a *Tyr*:RAS^+^/Ink4a/Arf^-/-^ transgenic mouse model, in which animals developed UM. In melanocytes, *HRAS* is driven under the control of tyrosinase promoter, in absence of Ink4a/Arf tumour suppressor. Spontaneous ocular and cutaneous melanomas occurred in 15.7% and 50.4% of cases, respectively. All UM tumours that developed were of choroidal origin, with none arising from retinal pigment epithelium. Morphologically, tumours resembled that of human UM tumours, having a spindle-like morphology. While many of the tumours extended to the subretinal space, no cases of metastasis arose in this model ^61^. However, the validity of this transgenic in modelling UM may raise concern, due to the co-occurrence of cutaneous melanoma in approximately 50% of cases. Furthermore, this model is based on overexpression of mutant *HRAS*, while *RAS* mutations are not thought to occur in UM 62.

Schiffner* et al*. developed a model of spontaneous UM using a Tg (*dct*:Grm1) transgenic mouse. Mouse dopachrome tautomerase (dct) promoter was cloned upstream of metabotropic glutamate receptor 1 (Grm1) transgene, to drive melanocyte-specific expression of Grm1. UM-like melanocytic tumours were observed intraocularly in the choroid and ciliary body, while cutaneous tumours occurred in the tails, ears, and anus. A range of tumour severity was seen in replicates of this model 63. While this model gives rise to UM tumours, the evidence supporting an established role of Grm1, or elevated expression, is lacking. However, with further research, Grm1 may present a novel therapeutic target in the treatment of UM.

More recently, Huang et al. successfully replicated genetic mutations of UM in a model overexpressing oncogenic *GNAQ*^Q209L^ allele under the control of the Rosa26 promoter. After only 3 months, UM tumours occurred, driven by *GNAQ*^Q209L^. Furthermore, most animals developed tumours in the lungs, which is a common site of metastases in human UM, aside from the liver. However, these lesions may have been of primary or metastatic origin. Yap activation, which occurs downstream of *GNAQ*, was detected in choroidal growths. Other results of this mutation were shown as central nervous system melanomas 64. This study presents a strong oncogenic potential of *GNAQ*^Q209L^ in UM tumorigenesis.

## Zebrafish Models of Uveal Melanoma

Zebrafish is a widely used invertebrate animal model in the preclinical setting, posing many beneficial characteristics, such as sharing large genomic similarities with humans. As much as 70% of human genes have similar zebrafish orthologs 67. In fact, much of the genes associated with diseases in humans have corresponding zebrafish counterparts, including highly conserved tumour suppressor genes, cell-cycle genes, and oncogenic signalling, such as tp53 68, cyclins, cdks and cdk inhibitors 69, and oncogenic Notch signalling 70, making for an extremely clinically relevant animal model. Furthermore, zebrafish models retain similarities with tumours on histological, genomic and gene expression levels 71; therefore, presenting as a suitable animal model in the context of UM.

Alterations are primarily applied to the developing embryo, where ex-utero development allows for convenient manipulation. The highly adaptive, immature immune system of the early developing embryo permits the administration and integration of tumour grafts at these early embryonic stages 72. This circumvents the issue of immunosuppression, required by most other xenograft models 73,74.

Additionally, the transparent nature of the fish embryos aids in tumour visualisation and live imaging of internal processes 75. Xenograft zebrafish models, involving the transplantation of human tissue or cells into the recipient animal model, are an accessible, time and cost-effective method of performing *in vivo* screens for anti-cancer agents for UM. In a study by van der Ent et al., a panel of 5 UM cell lines were injected into 2-day old zebrafish embryo yolk sacs and fixed 6-days post-injection. Drug administration with individual treatment of either dasatinib, quisinostat or MLN-4924, was performed 1-day post injection in selected animals, to screen for therapeutic effects. The models displayed a phenotypic reflection of the cell lines' characteristics, consistent with clinical behaviour and underlying genetics of the UM patients of origin. OMM2.3, originating from a metastasis and harbouring the *BAP1* mutation, showed increased onset of migrating tumour cells in zebrafish, compared to its primary tumour counterpart, MEL270. Src-inhibitor dasatinib caused reduced proliferation and migration in the high Src-expressing 92.1 cell line, while having little effect on low Src-expressing OMM-2.3 cells. Additionally, quinostat, a histone deacetylase inhibitor, and MLN-4924, a neddylation pathway inhibitor, prevented proliferation and migration in both 92.1 and OMM-2.3 cell lines 76. Studies as such can provide a means of predicting patient response to treatment, identifying optimal therapeutics for UM cases of a specific disease class. This study effectively demonstrates the relevance of this model in drug screening and drug discovery.

Zebrafish UM xenografts are also beneficial in studying metastatic processes. Fornabaio et al., developed zebrafish xenografts from metastatic cell lines, OMM-2.3 and OMM-2.5, to investigate angiotropism and extravascular migratory metastasis in UM. Interactions between xenograft cancer cells and the external surfaces of vasculature were monitored via *in vivo* imaging techniques. Results of this study showed that UM cells migrated along vascular surfaces similarly for *in vivo* xenograft models, and *in vitro* endothelial tubule assays 50.

Zebrafish expressing human oncogenes can be generated using transgenesis, introducing a new phenotype to the developing zebrafish, which is also transmitted to offspring. Many transgenic zebrafish models have successfully been generated, for cancers including leukaemia and lymphoma, allowing for reverse genetic screening 77,78. The first transgenic zebrafish model of UM was generated by Rose et al. This model displayed spontaneous development of UM from overexpression of a human UM oncogene, transgenically. Tg (mitfa:BRAF V600E);p53-/-;mitfa-/- transgenic embryos were injected with Tol2 transposase mRNA and transposon-based miniCoopR plasmid, overexpressing oncogenic *GNA11* Q209L in rescued melanocytes 79. *HOXB7* overexpression was observed in the induction of UM in zebrafish melanocytes. There is no past evidence for a role of *HOXB7* in UM tumorigenesis, but *HOXB7* has been shown to be overexpressed in other cancers, including cutaneous melanoma 80, and could contribute to a pro-proliferative state in the presence of the tumour microenvironment. Furthermore, in the absence of melanocyte-restricted stable expression of the BRAFV600E transgene, no tumours were observed, which itself is a limitation to this model. In human UM, *BRAF* mutations are uncommon, meaning that this model may not reflect the genetics of human UM 81,82. Another example of the use of transgenesis in zebrafish UM research, was that of Mouti et al. using a Tg (cryaa:Venus,mitfa:gnaq_Q209P);tp53 M214K transgenic zebrafish model. In this study, two transgenics were successfully generated, representing benign and malignant stages of UM development, and documenting the first *in vivo* characterisation of *GNAQ*^Q209P^ as a molecular driver in UM. *GNAQ*^Q209P^, an oncogenic form of *GNAQ*, was expressed under the control of the mitfa promoter, which allowed selective targeting of melanocytes. This resulted in hyperproliferation of uveal melanocytes, and not cutaneous melanocytes, in the model, which reflects the clinical consequences of *GNAQ* mutations in human UM. Moreover, UM tumours derived from this model show that ERK1/2-MAPK signalling is weakly activated downstream of mutated *GNAQ* in UM, which is consistent with results from similar studies using established human UM cells. Interestingly, the addition of tp53 inactivation to this model was sufficient to introduce malignant transformation of uveal melanocytes, reflecting human UM, where cases harbouring *GNAQ/11* are generally benign 83,84. Similarly, a study by Perez et al. focused on zebrafish models expressing oncogenic forms of *GNAQ* and *GNA11* (*GNAQ/11*^Q209L^). As in previous publications, combination of these driver mutations with mutant tp53 saw the development of ocular melanomas which were primarily choroidal, and shared molecular and histological characteristics with human UM. Expectedly, nuclear YAP localisation was noted, mirroring events in human UM of the same mutational burden. Tumours were also reported at cutaneous and internal sites in this model 85. However, evidence of melanocytic alterations was also observed in the presence of either *GNAQ/11*^Q209L^ mutations alone, therefore, indicating that such mutations may be sufficient to promote early tumorigenic processes *in vivo.* Despite such findings, it is likely that there is a plethora of mutational contributors that may depict the oncogenic strength of *GNAQ/11*^Q209L^. Ju et al. generated a transgenic zebrafish line expressing EGFP fusion protein of an activated zebrafish Smoothened (Smoa1-EGFP) using the Gal4VP16-UAS binary transgenic expression approach. While expression of Smao1-EGFP alone did not result in tumour formation, co-expression of constitutively active human AKT1, lead to ocular melanomas presenting in the retina, among other tumour types, suggesting a role for Smao1 in zebrafish tumorigenesis. Moreover, activation of the PI3K-AKT pathway was indicated by elevated levels of phosphorylated AKT 86. Such findings propose that tumorigenesis can occur as a result of co-activation of hedgehog and AKT pathways in this model. This study demonstrates the utility of transgenic zebrafish models in examining the dynamics of oncogenic signalling pathways.

There is a clear potential for the use of transgenic zebrafish models in capturing the genetics of UM, and serving as an effective preclinical model of UM. Notably, cell lines from this model, as described by Mouti et al. were similar to human UM cell lines when comparing genetic and drug response characteristics, suggesting parallels between this animal model of disease, and the human correlate 83,84. Such models are, therefore, useful in exploring novel genomic signatures in tumorigenesis, to further understand the complexity of the tumour microenvironment and its potential therapeutic targets. However, drug screening in zebrafish has several limitations. While there is a high degree of conservation between zebrafish and humans, differences remain, which could give rise to non-translatable chemical and drug screening hits.

### Preclinical modelling and targeted therapy clinical trials in UM

Building on preclinical translational research to inform clinical studies is of high scientific relevance for the development of new therapeutic targets for UM. Preclinical models need to provide data demonstrating the efficacy of targeted therapeutics based on the (epi)/genomic and transcriptional landscape of the tumour. Barriers to the implementation and efficacy of clinical studies may relate to the (epi)/genetic and transcriptional heterogeneity of the original UM, confounded by the lack of appropriate preclinical models used to inform clinical studies.

Mutations commonly arising in *GNAQ* and *GNA11* in UM can lead to increased activation of the Ras/Raf/MEK/ERK pathway, which is involved in the control of cell proliferation, survival, and differentiation 87. Anomalies affecting this pathway are integral in the development of UM, therefore, targeting this pathway is a common point of therapeutic intervention. Table 3 outlines the results of completed targeted therapy clinical trials in UM and links associated preclinical studies and models. Clinical trials of MEK inhibitor selumetinib (phase III) in mUM, while proving safe, failed to demonstrate a significant improvement in progression-free survival (PFS) 88,89. Despite a small number of experiments showing anti-tumour activity and reduction in tumour volume, selumetinib monotherapy, or in combination with dacarbazine (DTIC) or AKT inhibitor, did not cause a significant objective response (OR) in the chosen experimental models 90,91. The MEK 1/2 inhibitor trametinib alone did not induce anti-proliferative effects in UM cell lines 92, *in vitro* studies in combination with ABT263 were synergistic, however this combination did not show anti-tumoral effects in PDX *in vivo* models 93. Furthermore, phase II clinical trials of trametinib in combination with AKT inhibitor GSK2141795 did not improve PFS in advanced UM 94.

Another example of indirect targeting of *GNAQ* and *GNA11* signalling is via protein kinase C (PKC) inhibition. PKC family proteins are involved in a number of pathways, including those regulating cell proliferation, differentiation, survival and death 95. PKC signalling alterations can be co-expressed with *GNA11, GNAQ*, and less commonly, *PLCβ4* mutations, all of which signal upstream of PKC. Thus, inhibition of PKC is a rational point of therapeutic intervention. Currently AEB071 and LXS196, PKC inhibitors, are in clinical trials both alone and in combination with other drugs 96,97. Preliminary data suggests clinical activity of AEB071 and manageable toxicity at multiple dose levels, with evidence of PKC inhibition in patients with UM 96. Corresponding *in vitro* studies using primary and mUM and cutaneous melanoma cell lines, suggest that AEB071-induced growth suppression of *GNAQ* mutant cells is associated with pronounced G1 arrest, and induction of apoptosis 98. While AEB071 inhibits PKC signalling *in vivo* in allograft mouse models, compensatory mechanisms prevent suppression of the MAP-kinase pathway. However, combinations of PKC and MEK inhibition were efficacious *in vitro* and *in vivo* causing marked tumor regression in a UM xenograft model 99.

A recent phase II clinical trial carried out by Buchbinder et al. investigated the anti-tumour capacity of ulixertinib (BVD-523) (ERK inhibitor) in mUM 100. While preclinical *in vitro* studies by Germann et al. involving ulixertinib, exhibited anti-proliferative and pro-caspase activity, such studies were carried out on a variety of adenocarcinoma cell lines and malignant melanoma cell lines, and not UM cell lines 101. Thus, the clinical translation of such models in the context of UM is unclear. Furthermore, a mouse model with a BRAF-mutated melanoma cell line xenograft was used as an *in vivo* model. Although ulixertinib demonstrated tumour growth inhibition and regression, as well as anti-proliferative synergism with BRAF inhibition, the absence of plausible UM models is questionable. Despite some parallels, in CM genetic aberrations and that of UM, there is a stark difference in each disease's genetic landscape, including the lack of BRAF mutations in UM, a hallmark of CM. Unfortunately, preliminary findings did not translate to the clinic. Progression as far as phase II revealed that ERK inhibition with ulixertinib was unsuccessful as a treatment for mUM, with no apparent improvement in PFS and OS observed 100. This negative result is likely owing to the use of unsuitable preclinical UM models, which may have proved poor predictors of the true clinical outcome.

Receptor tyrosine kinases (RTKs) have also been implicated in tumorigenesis. RTKs are involved in the processes of cell-cell communication, cell growth and differentiation, among many, and are associated with growth and malignancy in cancers 102. Sunitinib is a multi-targeted receptor targeted (RTK) kinase inhibitor against VEGFR, FMS-like tyrosine kinase 3 (FLT-3), C-KIT and platelet-derived growth factor receptor (PDGFR). A randomised phase II clinical trial combining sunitinib or DTIC was carried out on mUM patients, and found no improvement in PFS or OS, and lacked significant clinical activity 103. The *in vitro* effects of sunitinib have been studied but in non-UM preclinical models. Studies in neuroblastoma cell lines and acral melanoma cell lines found sunitinib inhibited tumour cell proliferation and phosphorylation of VEGFRs. Similarly, *in vivo* sunitinib caused a reduction of tumour growth, angiogenesis, and metastasis of neuroblastoma models *in vivo* 104.

Vascular endothelial growth factor (VEGF) is another common target in cancer treatments. VEGF signalling promotes tumour survival as it plays a crucial role in tumour angiogenesis, and therefore, inhibitors of this pathway can have potent anti-tumour effects. Multi-kinase inhibitors such as cediranib and sorafenib, have been investigated as potential therapies for the treatment of UM. In a phase II clinical trial by McWhirter et al. cediranib was administered continuously on a 28-day cycle to mUM patients. While some patients had a stable disease following treatment, in the vast majority of cases, disease progression occurred over a short period of time. Moreover, the lack of OR observed in this study group indicates that cediranib may not be efficacious as a single agent treatment in UM 105. Preclinical assessment was carried out using non-UM glioblastoma cell lines and mice with RENCA cell xenografts. *In vitro* studies showed that cediranib inhibits cellular migration and invasion, while *in vivo,* cediranib caused a significant reduction in tumour volume over a range of drug treatment periods 106,107. Sorafenib has also been examined clinically as a mUM therapy. Phase II clinical trials were carried out on sorafenib in combination with carboplatin and paclitaxel in patients with mUM (SWOG S0512). Minor tumour responses and disease stability were observed in some patients, however, due to the poor OR, clinical examination was not further progressed using sorafenib and the combination 108. Pre-clinical non-UM cell lines such as those derived from hepatocellular carcinoma demonstrated anti-cancer properties to sorafenib 109. Sorafenib has been tested in a xenograft model in which uveal melanoma cell line 92.1 was dorsally injected subcutaneously; this study found that sorafenib inhibited of tumour growth (*p≤*0.0035) and fewer metastases after sorafenib treatment were observed (33% *vs.* 60%) 110. However, this did not translate into clinical efficacy of sorafenib for the treatment of UM.

## Conclusion

**Figure 1:**
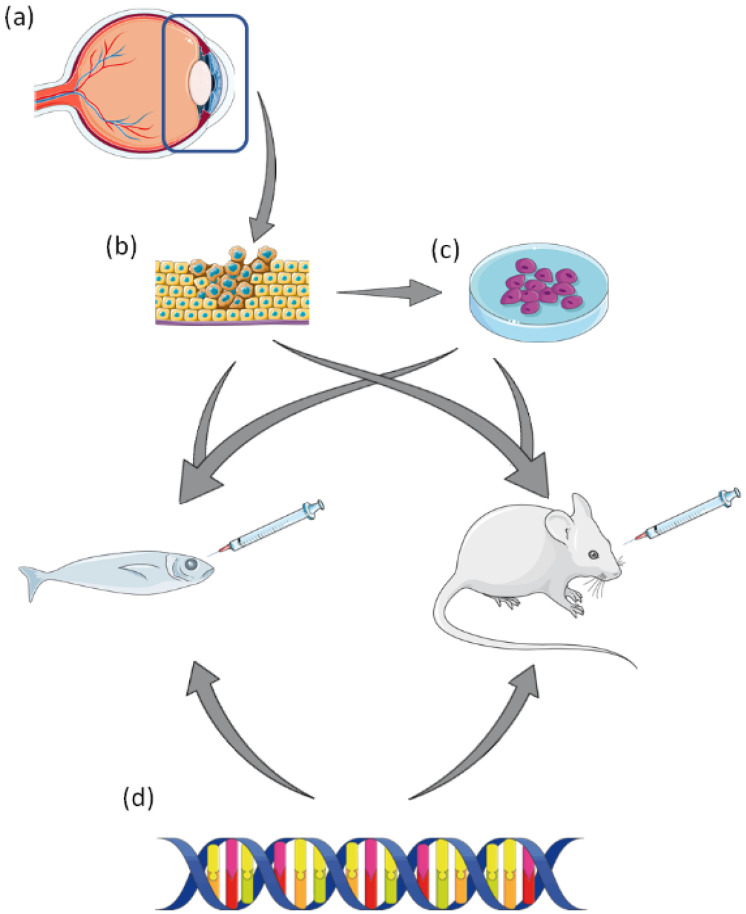
** A summary of pre-clinical laboratory models of Uveal Melanoma.** (a) Tumour resection of affected eye. (b) Resected tumour can be used to generate UM cell lines, or patient derived xenograft (PDX) in animal models. (c) Established UM cell lines can be used to generate xenograft animal models. (d) UM animal models can be created by genetic manipulation via transgenesis. This figure was created using Servier Medical Art templates, which have been modified. These images are licensed under a Creative Commons Attribution 3.0 Unported License; https://smart.servier.com.

Preclinical modelling and drug screening in established cell lines and animal models are an integral part in bridging the translational gap (Figure 1), providing a roadmap for regulatory drug development and approval. While the current models in place for studying UM have proved vital in gaining a greater understanding of the disease, as with most preclinical models - they do not fully recapitulate the *in vivo* situation, and to date, no UM preclinical studies have predicted effective translation to the clinic. Therefore, better strategies are needed to improve the translational relevance of preclinical to clinical translational research in UM.

## Future directions

Advancements in *in vitro* cellular models such as 3D organoids have the potential to create a more accurate representation of how tumours grow and interact with surrounding cells and tissues. Preclinical models that reflect their original cancer help to make preclinical data more reproducible and translatable to the clinic. Therefore, utilisation of preclinical model systems which fully recapitulate the human *in vivo* situation may enhance the translational impact of experimental data, and inform future strategies for clinical impact.

## Figures and Tables

**Table 1:** Summarised established uveal melanoma cell lines

Cell line [Refs]	Origin	Sex	Morphology	Pop. doubling time	GNAQ	GNA11	BAP1 mut	BAP1 protein	EIF1AX	SF3B1	Cytogenetics					
											Chr 1	Chr 3	Chr 6	Chr 8	Chr 16	Other
92.1 36,37	Met	F	Mixed	38 h	Q209L (626 A>T)	WT	WT	Y	c.17G/A	Yn(C1793 C>T)		Disomy 3	6p+	8q+		der (X) t (X; 6)(q28; p11),+8
MEL-202 36-38	Primary	F	Epithelioid	43 h	Q209L (626 A>T) R210K (629 G>A)	WT	Y	Y		c.1793c>T		Disomy 3	6q-, 6p+	8q+^41^		
MEL-285 36,39	Primary	F			WT	WT			WT	WT		Disomy 3, 3p26-	6q-	Disomy 8p, 8q+		
MEL-270 35,39	Primary	M			Q209P (626 A>C)	WT	Y	Y	WT	WT		Disomy 3, 3p24-, 3q21.2-3q24	6p+	Disomy 8q, 8+		
MEL-290 35,39	Primary	F	Epithelioid		WT	WT			WT	WT		Disomy 3, 3p26	Disomy 6	Disomy 8		
OMM-1 35,40	Subcut. met.	M	Mixed	34 h	WT	Q209L (626 A>T)	Y	Y	WT	WT		3+		8p-		4-, 7+, 9-, 11-, 12+, 15-, 17-, 20+, 21-
OMM-2.3 35,40	Liver met.	M			Q209P (626 A>C)		Y	Y	N/A	N/A						
OMM-2.5 35,40	Liver met.	M	Mixed	50 h	Q209P (626 A>C)		Y	Y								
MM28 41	PDX liver met.	M	Mixed	109 h		c.626A > T	c.1881C>A	N			1p-, 1q+	3q-	6q-, 6p+	8p-, 8q+	16-	
MM33 41	PDX skin met.		Spindle	91 h	c.626 a > C			Y	c.22G/A		1+		6q-, 6p+	8+	16+	
MM66 41	PDX liver met.		Mixed	80 h		c.626A > T	N	Y			1q+		6q-	8+		
MP38 41	Primary	M	Spindle	80 h	c.626 a > T		c.68-9_72 del	N				3q-		8+	16q-	
MP41 41	PDX primary	F	Mixed	41 h		c.626 a > A/T	N	Y			1p-, 1q+	3-	6q-, 6p+	8p-, 8q+	16-	
MP46 41	PDX primary	M	Mixed	110 h	c.626 a > T		N	N			1q+		6q-, 6p+	8p-, 8+	16q-	
MP65 41	Primary	F	Spindle	120 h		c.626A > T	c.1717del	N			1q+		6p+	8+		
UPMM-1 42-44	Primary	M	Mixed	100-150 h	R183Q (548 G>A)							3-				
UPMM-2 42-44	Primary		Spindle	150 h	Q209L (626 A>T)							3-				
UPMM-3 42-44	Primary	M	Epithelioid	100 h	p.Gln209Pro (c.626A>C)							3-				
UPMM-4 42-44	Primary	M		300 h	WT	WT										
UPMD-1 43	Primary			100 h		Q209L (626 A>T)						Disomy 3				
UPMD-2 43	Primary			150 h		Q209L (626 A>T)						Disomy 3				
UMT2 45		M		72 h	WT	Q209L (626 A>T)						Disomy 3				
UMT26 45		M		2000-3000 h (3-4 months)	WT	Q209L (626 A>T)										
UMT33 45		F		288 h	WT	Q209L (626 A>T)										
WM3618F 46	Lymph Node Met.	F	Melanocytic		Y		Y									
WM3772F 46	Lung Met	F	Clumpy pigmented		Y		Y									
MEL-20-06-039 47					Q209L (626 A>T)											
MEL-20-06-045 47					Q209P (626 A>C)^43,44^											
MEL-20-07-070 47						Q209L (626 A>T)										

Other UM cell lines in existence for which such information is unavailable include: 92.2, BB90-MEL, OMM2, OMM2.2, OMM2.6, OMM3, MU2, MU8, EOM-3, EOM-29, C918, M619, MuM-2B, MKT-BR, YUGLIDE, YUCRENA.

**Table 2:** Summarised mouse xenograft models and GEMMs

PDX Model	Phenotype	Limitations	Reference
MP34, MP38, MP41, MP42, MP46, MP47, MP55, MP71, MP77, and MP80	Original chromosome 3 status maintained in xenograft in the case of disomy, heterozygosity and monosomy	Where original tumours were isodisomic for chromosome 3, corresponding xenografts harboured monosomy 3 anomaly	41,54,58
6 Liver metastases successfully grafted 3 times	Key mutational and histological characteristics (CNV, immunohistochemical melanoma markers, ratio of Ki67 positive cells) of original tumour maintained in xenograft tumour	Orthotopic hepatic transplantation causes difficulties in monitoring tumour growth (CT scan is required)	55
ØPI-204	Models human uveal malignant melanoma; transplanted cells retained morphological similarities with the primary tumour; immunohistochemically representative of malignant melanoma	Transplanted cells stained positive for vimentin, unlike the primary tumour	65
**GEMM**			
*Tyr*:RAS+/*Ink4a*/*Arf*-/-	Spontaneous ocular and cutaneous melanomas, all choroidal origin; morphologically similar to human UM	No metastases; 50% of mice developed cutaneous melanoma	61
*Tg* (*dct*:*Grm1*)	Spontaneous choroidal and ciliary body uveal melanomas; cutaneous melanomas	Cutaneous melanoma present	60
*Rosa26-LSL-GNA11^Q209L^/+;Tyr-CreER^T2^/+* *Rosa26-LSL-GNA11^Q209L^; Bap1lox/lox; Tyr-CreER^T2^/+*	Spontaneous uveal, cutaneous and leptomeningeal melanomas; evidence of potential metastases in axillary lymph nodes and lungs	Cutaneous and leptomeningeal melanomas; no evidence of liver lesions	60
	Accelerated rate of disease compared with above (*Rosa26-LSL-GNA11^Q209L^/+;Tyr-CreER^T2^/+*); Bap1 deletion contributed to growth of cutaneous melanoma and increased mortality rates		60,66
*Rosa*26-floxed stopGNAQ^Q209L^/+; *Mitf*-cre/+	Spontaneous uveal melanoma with occasional cutaneous,leptomeningeal and vestibular melanocytic lesions; evidence of lung metastasis	Cutaneous and leptomeningeal melanomas; no evidence of liver lesions	64
*Dct*-*rt*TA/+; *tet-HA-GNAQ*^Q209L^/+; *p16p19^KO^*	Spontaneous cutaneous melanoma in >50% animals	No uveal lesions reported; cutaneous melanomas present	58

**Table 3:** Completed clinical trials of targeted therapies and matched laboratory preclinical studies in uveal melanoma

Targeted agent	Preclinical models	Preclinical results		Clinical Trial				Refs
		*In vitro*	*In vivo*	Trial	Study design	Results	Findings/Conclusion	
Selumetinib (MEK 1/2 inhibitor)	Selumetinib and DTIC alone and in combination in 6 UM cell lines; MP38, MP41, MP46, MP65 (primary); MM28, MM66 (liver mets). Selumetinib and AKT inhibitor alone and in combination with Mel202, Mel270, Mel290, 92.1, OCM1A (primary); Omm1.3 (liver mets). *In vivo*: PDX-derived UM tumours	Selumetinib alone showed response in 3 out of 6 CL -Weak non-sig synergistic effect of combination in 5 of 6 cell lines -Combinatorial Selumetinib and AKTi treatments inhibited cell viability more effectively than monotherapy in GNAQ and BRAF-mutant cells	Selumetinib alone showed no sig TGI in 2 of 3 PDX models. OR = 18% DTIC alone showed no sig TGI in 2 of 3 PDX models OR = 15% Combination was not more effective than either drugs alone OR = 28%	Phase III SUMIT (n=129) NCT01974752	mUM, no prior systemic therapy randomly assigned selumetinib (75 mg twice daily) plus DTIC (1,000 mg/m^2^ intravenously on day 1 of every 21-day cycle) (n=97) or placebo plus DTIC (n=32)	Selumetinib plus DTIC arm 85% achieved PFS event vs 75% placebo (median 2.8 v 1.8 mo) HR PFS 0.78 (95% CI, 0.48 to 1.27; two-sided P = 0.32). ORR 3% with selumetinib plus DTIC and 0% with placebo plus dacarbazine (two-sided P = 0.36)	Combination of selumetinib plus DTIC had a tolerable safety profile but did not significantly improve PFS compared with placebo plus DTIC.	88-91
Trametinib (MEK 1/2 inhibitor)	MET CL: MM28 (liver metastasis), MM66, OMM1 (subcutaneous met), OMM2.3 and OMM2.5 (liver metastasis) Primary CL: MP38, MP46, MP65 *In vivo*: Xenografted SCID female mice	1/11 UM CL sensitive to trametinib Combination of trametinib and ABT263 (Bcl-2/XL/W inhibitor) displayed the best rank synergistic combination in 8 UM CL.	*In vivo* efficacy of ABT263 + trametinib in 6 UM PDX found trametinib alone had the best anti-tumour effect, but in combination with ABT263 did not show any additive effect.	Phase II (n=39) NCT01979523	Advanced UM patients, no prior systemic or liver-directed therapy were randomized to one of two arms stratified by liver disease and LDH: trametinib 2mg daily (Arm A, 18 pts) or trametinib 1.5mg + GSK2141795 (AKT inhibitor) 50mg daily (Arm B, 21 pts).	Partial response was observed in each arm. No difference in median PFS between Arm B and Arm A (Median PFS trametinib + GSK2141795 was 7.9 wk). All pts had ≥1 adverse event (AE).	The addition of GSK2141795 to trametinib did not improve PFS.	92-94
AEB071 (PKC inhibitor)	Panel of 7 UM cell lines with GNAQ mutations. MET CL: OMM-1.3 OCM1. Cutaneous Melanoma CL: OCM3 Primary CL: C918 MEL285, MEL202, 92.1 *In vivo*: Allograft mouse model (GNAQ^Q209L^ were injected into the flanks of c57/Bl6 mouse)	AEB071 induced growth suppression of GNAQ mutant cells, with pronounced G1 arrest and induction of apoptosis *in vitro.* 7/11 UM CL sensitive/intermediately sensitive	The mice received 120 mg/kg AEB071 (n=9) or vehicle control (n=9) x3/day for 3 weeks by oral gavage. AEB071 significantly inhibits GNAQ^Q209L^- mediated tumour growth *in vivo* in combination with MEK inhibitor.	Phase I (n=153) NCT01430416 Phase Ib/II (n=38) NCT01801358 NCT02273219	mUM dose escalation and estimation of the maximum tolerated dose (MTD). 118 pts received AEB071 at total daily doses of 450-1400 mg either BID or TID.	Dose-limiting Toxicities (DLTs) were observed in 12 pts (11%) at doses ≥800 mg/day total. 55/118 (47%) pts achieved SD and progression-free survival (PFS) ranging from <1-57 weeks and with a median PFS of 15.4 weeks (95% CI 8.3-15.7).	Preliminary data suggests clinical activity of AEB071 and manageable toxicity at multiple dose levels, with evidence of PKC inhibition in patients with mUM.	96,98,99
Ulixertinib (BVD-523); (ERK inhibitor)	No uveal melanoma cell lines used RKO, SW480, HCT116, Colo205, HT-29 (colon/colorectal), A375 (cutaneous melanoma), MIAPaCa-2 (pancreas carcinoma), ZR-75-1 (breast ductal met site), AN3Ca (adenocarcinoma) and G-361 (malignant melanoma) *In vivo*: female athymic nude mice, xenograft model	*In vitro* ulixertinib treatment resulted in reduced proliferation and enhanced caspase activity in sensitive cells.	*In vivo* ulixertinib showed dose-dependent growth inhibition and tumor regression. ulixertinib yielded synergistic antiproliferative effects in a BRAF^V600E^-mutant melanoma cell line xenograft model when used in combination with BRAF inhibition.	Phase II NCT03417739 (n=13)	A phase II study to determine the efficacy and safety of ulixertinib in patients with mUM.	Median time to progression 2.0 months (90% CI: 1.8-3.6 mos.). Median survival time 6.9 months (90%CI: 3.2 to 8.3 mos.).	ERK inhibition with ulixertinib did not demonstrate activity in patients with mUM. The toxicities observed in the study were consistent with what would be expected with MAPK pathway inhibition.	100,101
Sunitinib; (RTK inhibitor)	No uveal melanoma cell lines Neuroblastoma cell lines: SK-N-BE(2), SH-SY5Y, LAN-5, NUB-7 cells, NB12, NB25, and NB88R2. *In vivo*: NB xenograft models, SK-N-BE(2) cells or NB12 cells injected subcutaneously into the groin fat pad of the NOD-SCID mice.	Neuroblastoma cell lines, SK-N-BE(2), NUB-7, SH-SY5Y, and LAN-5, were exposed to increasing concentrations of sunitinib for 72 hours and assayed. *In vitro* Sunitinib inhibits tumour cell proliferation and phosphorylation of VEGFRs.	Treatment with 20 mg/kg of sunitinib showed significant reduction (P < .05) in primary tumour growth. Sunitinib inhibits tumour growth, angiogenesis, and metastasis *in vivo*.	Phase II NCT01551459 (n=124)	Patients with mUM and no prior systemic therapy for advanced disease. They were randomized 1:1 to sunitinib (50mg daily for 28 days, followed by a 14-day break), or dacarbazine (1000 mg/m^2^ once every 21 days).	Overall response rates of 0% and 8% were observed in the sunitinib and dacarbazine arms; while stable disease was observed in 24% of pts on sunitinib, and 11% on DTIC. PFS and OS were not improved with sunitinib.	In these preliminary results, sunitinib did not exhibit significant clinical activity in mUM.	103,104
Cediranib (Multi-kinase inhibitor, VEGF)	No uveal melanoma cell lines Eight immortalized glioblastoma cell lines were used: SW1088, SW1783, U-87 MG, A172, SNB-19, GAMG, U251 and U373. *In vivo*: 6-to 8-week-old female BALB/c mice, injected with RENCA cells.	Cediranib IC50 of 1.71 ± 0.97 μM (range, 0.47-4.17 μM) Cediranib *in vitro* on glioblastoma cell lines inhibited cellular migration and invasion.	Mice were treated once daily with 5 mg/kg cediranib. Cediranib *in vivo* reduces primary tumour in mice in a dependent exposing time manner.	Phase II (n=24) NCT00243061	mUM cediranib was given on a continuous, oral once daily schedule of 45 mg, on a 28-day cycle.	Of the 17 patients evaluable for response, there was stable disease in 8 patients, and progressive disease in 9 patients, with no objective responses seen. Only 2 patients had stable disease ≥6 months.	Although 2 patients had stable disease at 6 months, the short median time to progression and lack of any objective responses indicate that single agent cediranib at this dose and schedule is not sufficiently active to warrant study continuation.	105-107
Sorafenib (Multi-kinase inhibitor, VEGF inhibitor)	No uveal melanoma cell lines Paediatric hepatocellular carcinoma cell lines: HC-AFW1, Huh7, HUH6, HepT1. *In vivo*: Xenograft model of HC-AFW1 cells	Tumour cell proliferation in the HC-AFW1 cell line was effectively inhibited by sorafenib.	Sorafenib was administered orally/day with a dosage of 60 mg/kg body weight. Treating mice bearing HC-AFW1-derived tumours with sorafenib only led to a moderate tumor growth inhibition.	Phase II NCT00329641 (n=25)	Patients with mUM who had received 0-1 prior systemic therapy were enrolled. Treatment included up to 6 cycles of carboplatin (AUC = 6) and paclitaxel (225 mg/m^2^ administered on day 1 plus sorafenib (400 mg PO twice daily), followed by sorafenib monotherapy until disease progression.	(ORR = 0% [95% CI: 0-14%]) This study was terminated at the initial stage. Tumour regression <30% in 11 of 24 (45%) patients. The median PFS was 4 months and the 6-month PFS was 29%. The median OS was 11 months.	The overall efficacy of CP plus sorafenib in mUM did not warrant further clinical testing when assessed by ORR, although minor tumour responses and stable disease were observed in some patients.	108-110

Abbreviations: CL, cell line; sig, significant; TGI, tumour growth inhibition; OR, overall response; ORR, objective response rate; met, metastatic; RTK, receptor tyrosine kinase; PFS, progression free survival; OS, overall survival.
